# NanoSIMS Analysis of Rare Earth Elements in Silicate Glass and Zircon: Implications for Partition Coefficients

**DOI:** 10.3389/fchem.2022.844953

**Published:** 2022-03-14

**Authors:** Lanlan Shi, Yuji Sano, Naoto Takahata, Mizuho Koike, Takuya Morita, Yuta Koyama, Takanori Kagoshima, Yuan Li, Sheng Xu, Congqiang Liu

**Affiliations:** ^1^ Atmosphere and Ocean Research Institute, The University of Tokyo, Kashiwa, Japan; ^2^ Guangzhou Institute of Geochemistry, Chinese Academy of Sciences, Guangzhou, China; ^3^ Center for Advanced Marine Core Research, Kochi University, Nanokoku, Japan; ^4^ Graduate School of Advanced Science and Engineering, Hiroshima University, Higashi-Hiroshima, Japan; ^5^ Graduate School of Science and Engineering, University of Toyama, Toyama, Japan; ^6^ Institute of Surface-Earth System Science, Tianjin University, Tianjin, China

**Keywords:** rare earth elements, NanoSIMS, silicate glass, zircon, partition coefficient

## Abstract

We have developed a method to analyze all rare earth elements in silicate glasses and zircon minerals using a high lateral resolution secondary ion mass spectrometer (NanoSIMS). A 2nA O^−^ primary beam was used to sputter a 7–8-μm diameter crater on the sample surface, and secondary positive ions were extracted for mass analysis using an accelerating voltage of 8 kV. A high mass resolving power of 9,400 at 10% peak height was attained to separate heavy REE from oxide of light REE. A multi-collector system combined with peak-jumping by magnetic field was adjusted to detect REEs and silicon-30 for calibration. Based on results of NIST SRM610 glass, sensitivities of REEs vary from 3 cps/ppm/nA of Lu to 13 cps/ppm/nA of Eu. Reproducibility of REE/Si ratios is better than 18% at 2σ. Secondary ion yields of REEs show positive relationships with their ionization potential of second valence. REEs of AS3, QGNG, and Torihama zircons were measured and calibrated against those of 91500 standard zircon. SIYs of REEs of zircon are identical to those of the glass standard. AS3 and QGNG data are generally consistent with those of previous work. Torihama REE data combined with the whole rock data provide partition coefficients of REEs between silicate melt and zircon. The relationship between these coefficients and ionic radius is explained by an elastic moduli model.

## Introduction

Rare earth elements (REEs) are an essential resource in industry (Kato et al., 2011; Hein et al., 2013) because they have unique optical and magnetic properties, and are necessary for high performance magnet and emitting phosphor of LED. In basic earth and planetary sciences their abundance patterns provide valuable information, such as fractional crystallization of magma, alteration of igneous rock, and origin of sedimentary carbonate (Henderson, 1984; Siklosy et al., 2009). There are several analytical methods of REEs for solid samples with detection limits of ppm-ppb in weight. They are instrumental neutron activation analysis (INAA) (Potts et al., 1973; Heaman et al., 1990), isotope dilution method by a thermal ionization mass spectrometer (ID-TIMS) (Kay and Gast, 1973; Fujimaki, 1986), inductively coupled plasma source mass spectrometer (ICP-MS) (Longerich et al., 1996; Chen et al., 1997), and secondary ion mass spectrometer (SIMS) (Shimizu and Richardson, 1987; Sano et al., 1999).

Among these analytical methods, both ICP-MS coupled with laser-ablation sampling technique (LA-ICP-MS) and SIMS instrument have a potential to detect REEs of sub-ppm level and a lateral spatial resolution of less than 50 μm scale. Generally, in trace element analysis, LA-ICP-MS has an advantage of easy operation, fast data acquisition, and less cost over SIMS. If a lateral resolution of less than 10 μm is required, however, SIMS is mostly applicable to the analysis with reasonable sensitivity (Zhang et al., 2016). There are two methods using SIMS to measure all REEs in earth and environmental samples. One is an energy filtering method to reduce the isobaric interference of light REE oxides onto the heavier REEs. This method would cause a significant loss of secondary ion transmission, down to two orders of magnitude, which results in inadequate sensitivity (Hoskin and Black, 2000). The other is a high mass resolution method to separate light REE oxides from heavier REEs. This method requires a large mass spectrometer with a high magnetic dispersion such as Cameca IMS-1280 and SHRIMP. The latter method is more sensitive than the former, while it takes a longer time to measure all REEs and matrix peak by a single ion counting detector with switching the magnetic field at least 17 times (Sano et al., 1999). We have developed a method to measure all REEs by a high lateral resolution secondary ion mass spectrometer (NanoSIMS) with a multi-ion collector system to reduce data acquisition time. Principally the method is similar to Zhang et al. (2016), while the mass-resolving power, sensitivity, and detection limit of REEs are assessed using a glass standard provided by a US national institute. We discuss the physico-chemical mechanism of secondary ion yields of REEs in this work.

Zircon (ZrSiO_4_) is an accessory mineral frequently occurring in crustal and felsic rocks, although its abundance is significantly minor in basaltic rocks. The REE concentrations and distributions in zircon are of great interest to geochemists who study the evolution of the Earth’s crust (Scherer et al., 2007). Because zircon is generally resistant to alteration, REEs in detrital zircon may keep original information of magma, even though a parent rock has long been lost. The REEs in 4.3–4.4 Ga zircon grains were used to argue for the presence of liquid ocean in Hadean era (Wilde et al., 2001). This is based on the partition coefficients of REEs between melt and zircon to reproduce the original magma compositions. In this work, we have applied the NanoSIMS analytical method of REEs to well-known standard zircons 91500, AS3, and QGNG. The precision and accuracy of the NanoSIMS method are assessed. Then, we have applied this method to estimate partition coefficients of REEs between silicate melt and zircon derived from a recent magmatic system in southwestern Japan. Zircon has eventually small inclusions of 1–10 μm in size. They are glass, apatite, and magnetite with different abundances of REEs (Sano et al., 2002). It is necessary to avoid the analyzing spot to overlap the inclusions. Therefore we need a high spatial resolution better than 10 μm.

## Experiment

### Sample Description and Preparation

In order to check the sensitivity and reproducibility of REE measurement, we have used a standard reference material “SRM610” produced and distributed by National Institute of Standards and Technology (NIST) in the USA. It is well-characterized silicate glass and all REE contents were reported fairly well (Rocholl et al., 1997; Jochum et al., 2011). For the REE analysis of zircon, we have used three international standard zircons, “91500,” “AS3,” and “QGNG.” The 91500 is a single-crystal zircon derived from Kuehl Lake in Ontario, Canada, and entered the mineralogical collection of Harvard University. Its trace element concentrations were well documented by Wiedenbeck et al. (2004) where REE contents were determined by both SIMS and LA-ICP-MS instruments of several institutions and data were compiled strictly. The AS3 are mostly sub-mm-size multi-crystal zircon grains extracted from the gabbroic anorthosite collected at the Duluth Complex, Minnesota, USA (Miller et al., 2002). Recently their REE concentrations were determined precisely and extensively by SHRIMP (Takehara et al., 2018). The QGNG is a multi-crystal zircon standard from Quartz-Gabbro-Norite-Gneiss from Cape Donnington, South Australia, which was used formally as a U-Pb dating standard by a SHRIMP (Sano et al., 2000). In order to calculate partition coefficients of REEs between silicate melt and zircon, we have used “Torihama” zircon extracted from the Torihama dacite pyroclastic pumice collected from southern Kyushu, Japan (Ui, 1971).

Small grains of SRM610 glass (approximately .5 mm × 1 mm) were mounted in an epoxy resin disc together with several grains of 91500 and AS3 zircons. They were polished to provide a flat surface for sputtering of secondary ions until their midsections were exposed. They were checked by SEM-EDS to locate inclusion-free homogeneous and non-crack regions. The Torihama zircons were mounted in the other disc together with a couple of QGNG zircons and SRM610 glass. They were polished until the mid-section was exposed. Both epoxy resin disks were coated by a thin gold plate 15–20 nm thick to prevent charging of the sample surface by the primary beam of the instrument.

### Analytical Procedure

Ion microprobe measurements of REEs were carried out using a NanoSIMS50 installed at the Atmosphere and Ocean Research Institute, The University of Tokyo. The residual pressure of secondary ion source chamber was measured as 5 × 10^−10^ Torr at the time of measurements, while those in magnetic analyzer and multi-ion collector housing were lower than 2 × 10^−8^ Torr. In a critical illumination mode which is the standard mode of the Cameca SIMS to control the shape and intensity of primary ions using a slit and apertures, a 2 nA mass filtered ^16^O^−^ primary beam was used to hit the sample surface perpendicularly at an energy of 16 keV and to sputter a 7–8 μm-diameter crater with a cone shape in depth. Secondary positive ions were extracted again perpendicularly by 8 kV for mass analysis. Before the actual analysis, the sample surface was rastered for 5 min in order to reduce the contribution of surface contaminant for 30 μm × 30 μm square. The entrance slit and each collector (exit) slits were set to 10 and 50 μm, respectively. In this condition, a mass-resolving power of 9,400 at the shoulder of 10%–90% peak height was attained to separate heavy REEs from the oxides of light REEs (the Cameca definition, see Supplementary Figure S1). No apparent isobaric interference was found in mass range from ^139^La to ^175^Lu in SRM610 glass. Zhang et al. (2016) reported the possible interferences of ^91^Zr^16^O_3_ on ^139^La and ^96^Zr^29^Si^16^O on ^141^Pr. We have carefully conducted measurements for the former interference using a deflector plate, while the latter was well resolved due to its lower required mass resolving power (approximately 5000). A transmission of approximately 10% ion was attained under this condition compared with a fully open entrance slit, which is ten times higher than the conventional energy filter method.

In order to present all REE abundances, we measured ^139^La, ^140^Ce, ^141^Pr, ^143^Nd, ^147^Sm, ^151^Eu, ^152^Sm, ^153^Eu, ^155^Gd, ^157^Gd, ^159^Tb, ^163^Dy, ^165^Ho, ^167^Er, ^169^Tm, ^173^Yb, ^174^Yb, and ^175^Lu together with a matrix peak of ^30^Si and background. For Sm, Eu, Gd, and Yb, two isotopes were measured to verify their isotopic compositions. All elements and/or isotopes were separated into six groups as B1 (^147^S and ^159^Tb), B2 (^151^Eu, ^163^Dy and ^173^Yb), B3 (^139^La, ^152^Sm and ^174^Yb), B4 (^30^Si, ^140^Ce, ^153^Eu, ^165^Ho and ^175^Lu), B5 (^141^Pr, ^155^Gd and ^167^Er), and B6 (^143^Nd, ^157^Gd, ^169^Tm and background), where the group was characterized by the same magnetic field of NanoSIMS. A multi-ion counting system was set up to measure all REEs where their physical positions were located as electron multiplier detector (EM)#1 = 222mm of turning radius for positive ions, EM#2 = 477 mm, EM#3 = 500 mm, EM#4 = 520 mm, and EM#5 = 535 mm. The magnet was cyclically peak-stepped from 0.3125T (to measure group B1 isotopes) to .3225T (B6), including .3166T (B2), .3176T (B3), .3186T (B4), and .3206T (B5) as shown in Table 1. Secondary ions were counted for 3 s for one cycle, resulting in a single scan through the spectrum taking ∼.5 min. The total counting time of each isotope was 150 s for fifty cycles and a complete run took approximately 30 min.

**TABLE 1:** A multi-collector system with peak-jumping by magnetic field to detect all rare earth elements (REEs) in silicate glass and zircon

	EM#1	#2	#3	#4	#5
Detector position	222 mm	477 mm	500 mm	520 mm	535 mm
MF = .3125T			Sm-147	Tb-159	
MF = .3166T			Eu-151	Dy-163	Yb-173
MF = .3176T		La-139	Sm-152		Yb-174
MF = .3186T	Si-30	Ce-140	Eu-153	Ho-165	Lu-175
MF = .3206T		Pr-141	Gd-155	Er-167	
MF = .3225T		Nd-143	Gd-157	Tm-169	BG

## Results

For the analysis of REEs in silicate samples by a SIMS, either precise Secondary Ion Yield (SIY) or Relative Sensitivity Factor (RSF, which is simply an inverse of SIY) is required to convert observed peak intensities into their concentrations. The SIY is calculated by measurements of standard samples with known amounts of REEs. Generally speaking, there is a variation of SIY due to the major chemical components of target samples (Deline et al., 1978), which is called by “matrix effect.” It is necessary to prepare a matrix matched standard before the actual analysis of REEs. At first, we carried out five spots measurements of NIST SRM610 glass where the ion beam of ^30^Si^+^ was used as internal standard. Table 2 lists the mass number, isotopes of REEs, their abundances relative to ^30^Si (A/^30^Si) in mol/mol, and observed A^+^/^30^Si^+^ ratios, where A denotes each REE isotopes and the REE abundances are from Jochum et al. (2011). The SIY was defined and calculated by (A^+^/^30^Si^+^)/(A/^30^Si). The error of SIY in Table 2 was estimated by a standard error based on the reproducibility of the A^+^/^30^Si^+^ ratios during repeated measurements. They vary from 10% of La to 18% of Sm at 2σ error. Sensitivities of REEs were independently determined under the same condition and changed from 3.0 cps/ppm/nA of Lu to 13 cps/ppm/nA of Eu with an average of 7.8 cps/ppm/nA, consistent with those reported by Zhang et al. (2016) using a NanoSIMS. The average of backgrounds was .67 ± .67 counts (1σ) for 150 s integration time. Then the detection limit of REE under the condition, 7–8 μm spot with 2 nA oxygen primary, is estimated by approximately 1 ppb due to 3σ background.

**TABLE 2:** Observed sensitivities and secondary ion yields of rare earth elements in SRM610 glass and 91500 zircon

Mass	Element	SRM610^a^ (ppm)	A/30Si mol/mol	A+/30Si+ obs	Sensitivity (cps/ppm/nA)	Secondary ion yield#	91500^b^ (ppm)	A/30Si (mol/mol)	A+/30Si+ obs	Sensitivity (cps/ppm/nA)	Secondary ion yield^c^
30	Si	10400					4,706				
139	La	400	8.29E-03	8.80E-02	7.58	10.61 ± 1.06	.0060	2.75E-07	2.85E-06	7.41	10.37 ± 4.87
140	Ce	373	7.69E-03	7.43E-02	6.90	9.65 ± 1.54	2.27	1.03E-04	8.01E-04	5.55	7.76 ± 1.71
141	Pr	423	8.65E-03	9.37E-02	7.73	10.83 ± 1.52	.024	1.09E-06	1.40E-05	9.21	12.89 ± 5.54
143	Nd	48.4	9.75E-04	1.22E-02	8.93	12.50 ± 1.87	.029	1.30E-06	1.43E-05	7.84	10.97 ± 3.84
147	Sm	61.8	1.21E-03	1.89E-02	11.16	15.63 ± 2.81	.075	1.47E-06	2.22E-05	10.78	15.09 ± 4.07
151	Eu	198	3.79E-03	6.89E-02	13.00	18.19 ± 2.55	.115	4.84E-06	9.29E-05	13.70	19.18 ± 4.80
152	Sm	110	2.09E-03	3.21E-02	10.99	15.38 ± 2.31	.134	5.60E-06	8.94E-05	11.40	15.96 ± 3.83
153	Eu	217	4.08E-03	7.47E-02	13.06	18.28 ± 2.56	.125	5.22E-06	1.04E-04	14.27	19.98 ± 5.00
155	Gd	62.3	1.16E-03	9.92E-03	6.11	8.55 ± 1.11	.327	1.35E-05	1.05E-04	5.60	7.84 ± 1.80
157	Gd	65.9	1.21E-03	1.03E-02	6.07	8.49 ± 1.10	.346	1.40E-05	1.05E-04	5.32	7.45 ± 1.64
159	Tb	413	7.49E-03	6.50E-02	6.19	8.67 ± 0.87	.860	3.45E-05	2.59E-04	5.37	7.52 ± 1.50
163	Dy	101	1.79E-03	1.84E-02	7.34	10.28 ± 1.44	2.94	1.15E-04	1.06E-03	6.61	9.25 ± 1.57
165	Ho	419	7.33E-03	6.24E-02	6.08	8.52 ± .85	4.84	1.87E-04	1.54E-03	5.87	8.21 ± 1.23
167	Er	95.7	1.65E-03	1.29E-02	5.57	7.80 ± 1.17	5.65	2.16E-04	1.71E-03	5.67	7.94 ± 1.43
169	Tm	415	7.08E-03	5.84E-02	5.88	8.24 ± 1.07	6.89	2.60E-04	2.36E-03	6.50	9.09 ± 1.27
173	Yb	67.8	1.13E-03	1.09E-02	6.87	9.61 ± 1.15	11.91	4.39E-04	5.52E-03	8.98	12.58 ± 1.64
174	Yb	134	2.22E-03	2.15E-02	6.91	9.67 ± 1.26	23.53	8.62E-04	1.10E-02	9.08	12.71 ± 1.78
175	Lu	396	6.54E-03	2.79E-02	3.05	4.27 ± 0.47	12.76	4.65E-04	1.97E-03	3.03	4.25 ± .55

a From Jochum et al. (2011).

b From Wiedenbeck et al. (2004).

c Error assigned to the SIY is 2σ.

We have measured all REEs of zircon 91500 standard for three spots by the same procedure as that of SRM610 glass. The average of observed data is listed in Table 2 following the glass data. In the analysis of zircon samples, light REEs such as La and Pr abundances were significantly small, usually less than 100 ppb. Thus their errors become larger. Overall errors of SIY vary from 13% of Lu to 47% of La at 2σ. Sensitivities of REEs were independently measured under the same condition and changed from 3.0 cps/ppm/nA of Lu to 17 cps/ppm/nA of Eu with an average of 8.3 cps/ppm/nA, which is very similar to that of glass standard. Then we analyzed REEs of AS3 for 1 spot and both QGNG and Torihama zircons for six spots. Resulted data were calibrated against those from the 91500 measurements. In practice, observed A^+^/^30^Si^+^ ratios were converted into REEs isotopic concentrations using SIY in Table 2. Then we calculated the elemental concentrations by their certificated isotopic compositions in La, Ce, Pr, Nd, Tb, Dy, Ho, Er, Tm, and Lu, while we took the average of concentrations given by two isotopes for Sm, Eu, Gd, and Yb. The REEs concentrations of AS3, QGNG, and Torihama zircons are listed in Table 3. The errors were estimated by quadrature combination of internal error (reproducibility of A^+^/^30^Si^+^ ratios) and calibration error using SIY of 91500 zircon.

**TABLE 3:** Rare earth elements concentrations in AS3 and QGNG zircons together with reference values

Element	AS3 (ppm)	Reference of AS3^a^ (ppm)	QGNG (ppm)	Reference of QGNG^b^ (ppm)
La	.250 ± .147	.096 ± .063	.042 ± .029	.088 ± .043
Ce	11.56 ± 3.62	7.69 ± 1.07	28.84 ± 6.11	39.20 ± 5.90
Pr	.544 ± 0.295	.578 ± .173	.260 ± .080	.370 ± .190
Nd	7.34 ± 3.07	7.60 ± 2.09	5.15 ± 1.33	5.00 ± 1.20
Sm	12.77 ± 4.45	9.21 ± 2.24	13.27 ± 4.09	8.20 ± 1.40
Eu	.399 ± .159	.331 ± .073	1.463 ± .496	.740 ± .230
Gd	42.7 ± 11.4	40.9 ± 8.5	44.4 ± 9.5	39.4 ± 8.2
Tb	15.63 ± 3.65	14.94 ± 3.18	15.42 ± 2.58	17.2 ± 3.7
Dy	165.6 ± 34.5	168.5 ± 30.0	198.3 ± 34.4	230.0 ± 43.0
Ho	53.2 ± 9.9	63.3 ± 10.7	55.1 ± 15.4	75.0 ± 17.0
Er	222.5 ± 45.8	261.0 ± 41.7	278.9 ± 66.8	340.0 ± 75.0
Tm	40.5 ± 6.7	54.2 ± 8.2	78.3 ± 19.6	75.0 ± 14.0
Yb	332.5 ± 50.8	408.6 ± 57.3	780.8 ± 201.6	620.0 ± 140.0
Lu	62.7 ± 10.3	89.6 ± 12.2	154.1 ± 37.0	109.0 ± 21.0

Errors are 2σ.

a From Takehara et al. (2018)

b From Sano et al. (2002)

## Discussion

### Secondary Ion Yields of REEs in Glass Standard

Observed secondary ion yields (SIYs) of REEs relative to ^30^Si in SRM610 glass vary significantly from 4.27 of Lu to 18.19 of Eu with an average of 10.84 (Table 2). There is no simple relationship between each REEs SIYs and their physico-chemical parameter such as density of solid, melting point, electronegativity, and ionic radius. Reed (1983) reported that SIYs of REEs relative to Ca^+^ in synthesized silicate glass varied from .12 of Lu to .81 of Eu using the AEI instrument, while Sano et al. (2002) presented that those relative to Si_2_O_3_^+^ in SRM610 glass changed from .0285 of Lu to .1048 of Eu by a SHRIMP. The elements and molecules used for normalization in the SIY calculations vary from study to study. In the present study, SIY is divided by Si, while Reed (1983) used Ca and Sano et al. (2002) took Si_2_O_3_. Therefore, it is difficult to directly compare the absolute value of SIY in this study with Reed (1983) and Sano et al. (2002). However, there are significant positive relationships among these SIYs measured by different analytical methods (see Supplementary Figure S2), suggesting a consistency of relative SIYs in three data sets. Linear regression of the SIYs between NanoSIMS and SHRIMP data gives a best fit of Y = −(0.67 ± 1.66) + (173 ± 32) X with 2σ error and a correlation coefficient, R = .989 (Supplementary Figure S2A). The best fit of SIYs between NanoSIMS and AEI data is expressed as Y = (1.9 ± 1.3) + (21 ± 4) X with 2σ error and R = 0.981 when the apparent outlier of Er is masked (Supplementary Figure S2B). There is a substantial offset of the Y-intercept, perhaps due to the high background of the AEI instrument. Considering these high correlation coefficients of SIYs from different analytical methods and/or sample matrix, there should be a governing physico-chemical mechanism of SIYs of REEs in silicate glasses.

**FIGURE 1:**
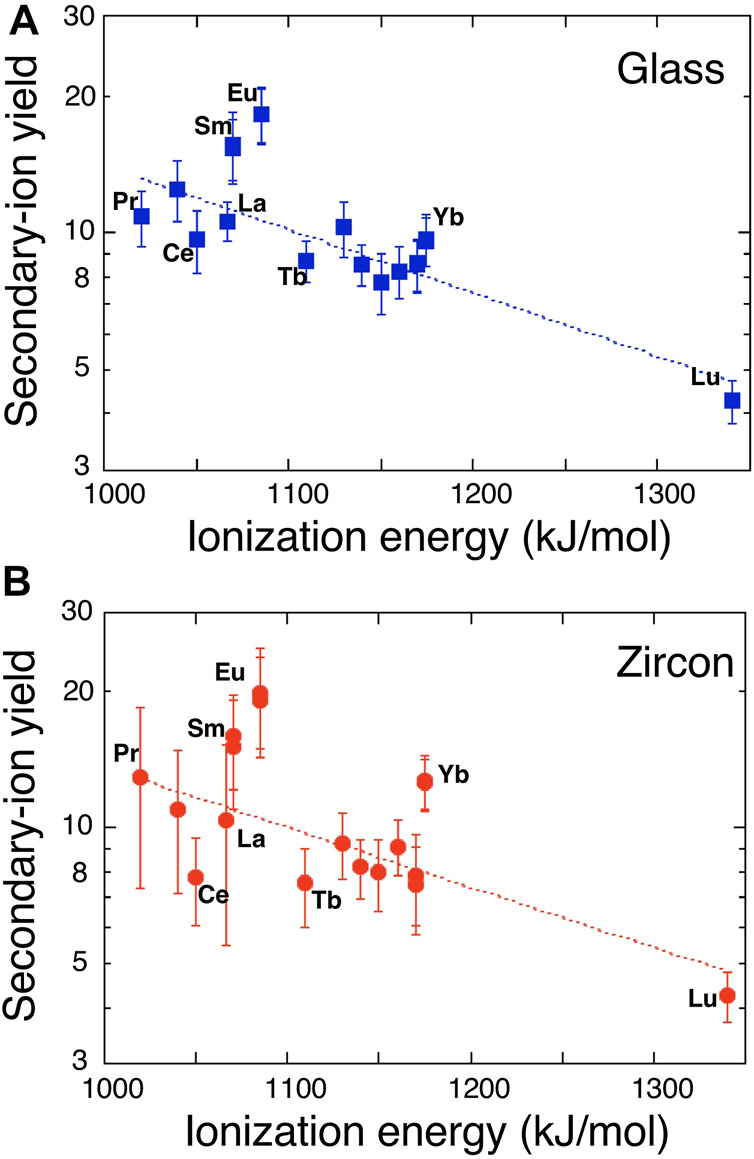
A correlation diagram between ionization energy of the second valence of rare earth elements and logarithm of secondary ion yields in **(A)** glass standard SRM610 and **(B)** zircon standard 91500. Error assigned to the symbol is 2σ. Dotted lines are the best fits of Eq. 1.

The local thermal equilibrium (LTE) model invented by Andersen and Hinthorne (1973) has been used widely for explaining the inter-element variations of SIYs during SIMS measurements. Based on the LTE model, Reed (1983) derived the formula of SIY as parameters of plasma temperature of ion production, partition function of the atoms, and ionization potential of the element. The formula would be modified into the following simple equation:

$$\text{SIY}=\left({\text{n}}_{\text{A}+}/{\text{n}}_{\text{Si}+}\right)/\left({\text{n}}_{\text{A}}/{\text{n}}_{\text{Si}}\right){=\text{const×e}}^{\left({\text{I}}_{\text{Si}}-{\text{I}}_{\text{A}}\right)/k\text{T}}$$ (1)

where T is the plasma temperature in K, n_A_ is the number of REE atoms or ions and I_A_ is the ionization potential of REE “A,” and *k* is the gas constant. If Eq. 1 is valid, there should be a linear correlation between I_A_ and logarithm of SIY. However, this is not the case. Reed (1983) reported that there is no negative relationship between the relative sensitivity of REE and the first valence I_A_, REE^+^. Instead, the relation was found between the sum of atomic and monoxide ion yields and I_A_ except for Lu, presumably because of competition between oxide and atomic ion formation during a primary beam bombardment on the sample surface (Morgan and Werner, 1977). Sano et al. (2002) documented that there is a clear negative relation between SIYs and the second valence of I_A_, REE^++^ including Lu, while no correlation existed with the first valence. Data of the present work are consistent with those of Reed (1983) and Sano et al. (2002). Again, there is no simple relationship between SIYs and the first valence. Figure 1A shows a correlation diagram between the ionization potential of second valence of REE^++^ and logarithm of REE SIYs. We calculated a linear regression of all data with I_Si_ = 1,577 kJ/mol (second valence of Si ionization potential) in Figure 1A where const and *k*T of Eq. 1 are 2.27 ± .48 and 314 ± 45, respectively (2σ error, R = .823, MSWD = 2.0). It is possible to calculate the plasma temperature to be 38,000 K that is much higher than values typically estimated in the source region of secondary ions by SIMS, perhaps caused by the effect of second valence. There are two apparent outliers of Eu and Sm with 3σ error off the trend. Anyway, we cannot show the physico-chemical mechanism to take the second valence of I_A_ instead of the first, probably due to the competitive ionization process of REE atom and oxide at the same time (Morgan and Werner, 1977). This is beyond the scope of the work and will be discussed in future study with SIY data of REE mono-oxides.

### Secondary Ion Yields of REEs in Zircon Standard

SIYs of REEs relative to ^30^Si in zircon standard, 91500, are listed in Table 2. They vary from 4.25 of Lu to 19.98 of Eu with an average of 11.5 where there is no simple relationship between the SIYs and physical or chemical characteristics of the element as stated above. Sano et al. (2002) reported that the SIYs relative to Si_2_O_3_^+^ in SL13 zircon changed from .0449 of Lu to .2699 of Sm by a SHRIMP. There is a positive correlation between these data (Supplementary Figure S3). Linear regression of the SIYs between NanoSIMS and SHRIMP data gives a best fit of Y = (1.7 ± 1.6) + (59 ± 16) X with 2σ error and R = .931. There is a very small offset of the Y-intercept. Most REEs except for Eu and Gd are consistent with the best fit line within 2σ error, suggesting the appropriate consistency of SIYs. Similar to SIYs of the glass standard, the LTE model is applied to zircon data. Again, there is no correlation between REE SIYs and the first valence I_A_. Figure 1B shows a relationship between the SIYs and the second valence I_A_. There is a clear negative correlation between I_A_ and logarithm of SIY, very similar to that of glass standard. The best fit of data into Eq. 1 by a linear regression presents that const and *k*T are 2.3 ± 0.5 and 326 ± 56, respectively (2σ error, R = .752, MSWD = 2.1). The calculated plasma temperature, 39,500 K, is much higher than that of the conventional estimate. There are three apparent outliers of Eu, Yb, and Ce with 3σ error off the trend, different from those of glass standard. It is noted that Eu is the outlier in both cases, probably due to chemical characteristics of Eu such as oxidation number in silicates and thus easy ionization by oxygen primary beam.

**FIGURE 2:**
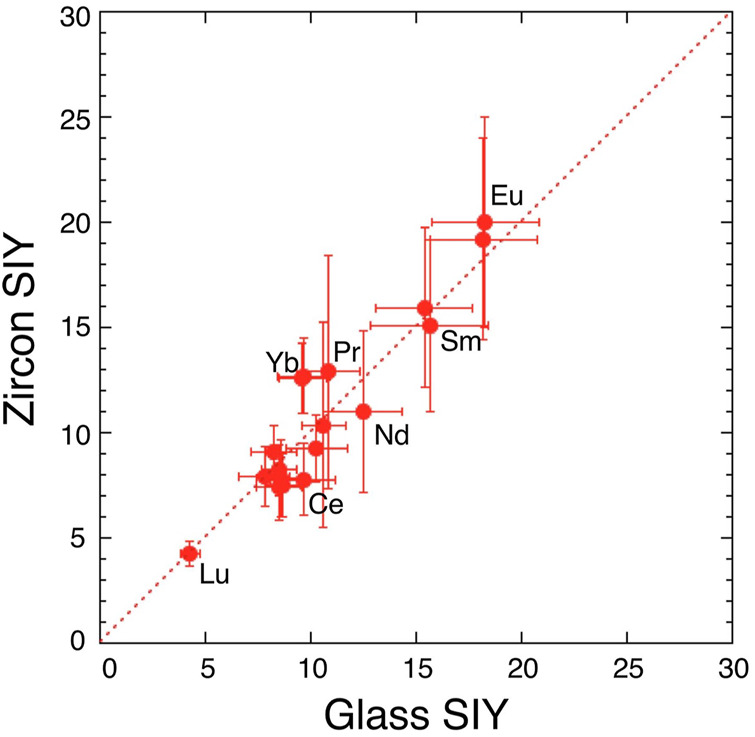
A correlation diagram between secondary ion yields (SIYs) of rare earth elements in glass standard SRM610 and those in zircon standard 91500. Error assigned to the symbol is 2σ. Dotted line shows consistent values of the glass and zircon SIY.

Estimated parameters of const and *k*T for zircon in the Eq. 1 are consistent with those of glass within experimental error, suggesting that the matrix effect on the ionization process is significantly small. This is verified by the direct comparison between two SIY data sets. Figure 2 shows a correlation diagram between SIYs of SRM610 glass and of 91500 zircon. There is a significant agreement of REE SIYs within 3σ error off the trend. Linear regression of all SIY data gives a best fit of Y = −(0.2 ± 0.9) + (1.03 ± 0.12) X with 2σ error and R = .934, where the best fit line is passing through zero point and the slope is approximately one. The result suggests that zircon SIYs are identical to glass SIYs (Figure 2) and the implication is that there is no matrix effect on SIYs between silicate glass and zircon. Similar results were reported by Sano et al. (2002) using a SHRIMP where they claimed that REE patterns (not absolute abundances) in zircon could be determined by SIMS with reference to a glass standard. It was difficult to obtain actual concentrations probably because the REE^+^ intensity was calibrated into an unstable matrix peak of Si_2_O_3_^+^ by SHRIMP. Zhang et al. (2016) claimed that there is a positive correlation between RSFs (inverse of SIYs) of REEs in glass and zircon. Our data are consistent with their claim, even though there is a difference of analytical procedure, that is, we took a conventional spot analysis, while Zhang et al. (2016) did a rastering of a small spot beam for 10 × 10 μm^2^.

In the present work, it is possible to measure all REE concentrations in zircon samples calibrated against a glass standard under the current experimental procedure. This is practically important when one would measure REEs in zircon.

### Accuracy of REE Measurements by NanoSIMS

In order to evaluate the accuracy of REEs measurement of zircon samples, we have analyzed two standard zircon samples, AS3 and QGNG, and calibrated against 91500 zircon. Table 3 lists REEs concentrations of AS3 and QGNG, where assigned errors are 2σ estimated by the combination of internal error of each measurement and error of SIYs obtained by analysis of 91500 zircon (see Table 2). We compare the REE data with those in the literature. Takehara et al. (2018) reported REE abundances of seventy-eight AS3 zircons using a SHRIMP installed at National Institute of Polar Research. Their data were classified into three groups based on transmitted light observations, BSE and CL images. Among them, type-C zircon was characterized by concordant U-Pb ages, low trace element concentrations such as Ca and Li, and bright BSE images, which suggests a primary origin of type-C without hydrothermal alteration. Our AS3 grain showed a bright BSE image, possibly belonging to type-C. We selected REE data of eight type-C zircons from Takehara et al. (2018) and calculated their averages and standard errors in order to compare with the present data.

**FIGURE 3:**
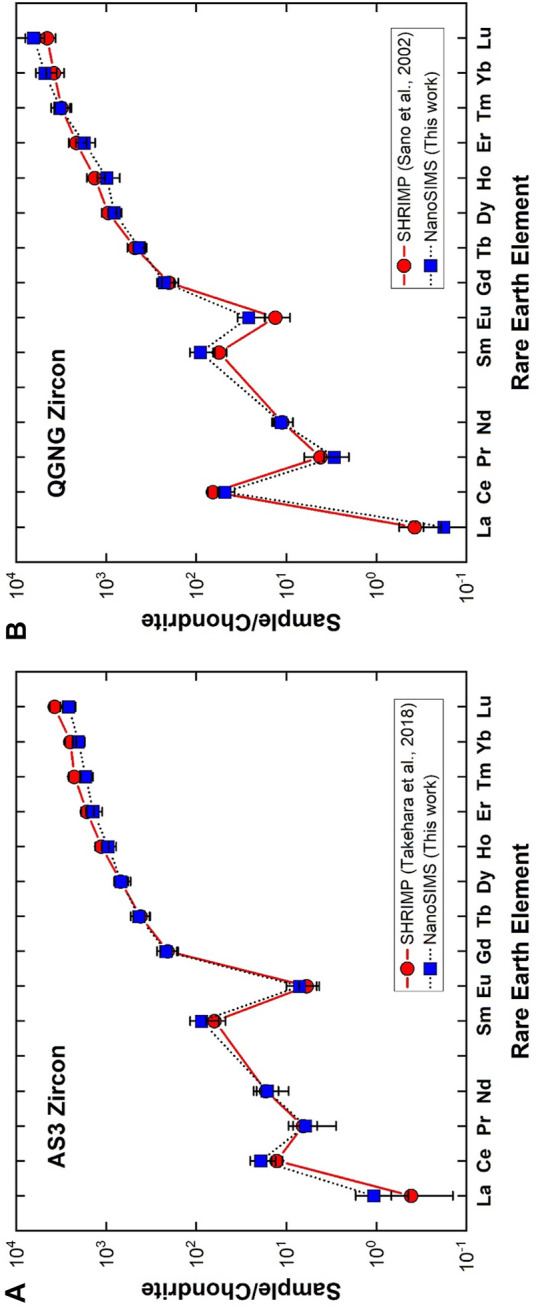
Chondrite normalized rare earth elemental abundances in **(A)** AS3 zircon and **(B)** QGNG zircon. Error assigned to the symbol is 2σ. A solid circle is from Takehara et al. (2018) measured by SHRIMP in NIPR, while a solid square indicates data of Sano et al. (2002) analyzed by SHRIMP in Hiroshima.

It is a conventional method to express REE abundances of silicate rocks by normalizing them to chondritic abundances and then to plot the logarithm of these normalized abundances in order of atomic number, which is called by a chondrite normalized REE-pattern or Masuda-Coryell diagram (Masuda, 1962; Coryell et al., 1963). Figure 3A shows REE-pattern of NanoSIMS measurement of AS3 zircon (solid squares; our data) together with an average of type-C zircons analyzed by a SHRIMP (solid circles; Takehara et al., 2018). Generally, the REE-pattern of zircon is characterized by a large fractionation between light REE and heavy REE with a progressive enrichment of the elements, positive Ce anomaly, and negative Eu anomaly. Our AS3 data together with those of type-C in the reference are consistent with these signatures. There is a significant agreement between two data sets within 2σ error except for the Lu value. To consider carefully the heavy REE values such as Er, Tm, Yb, and Lu, SHRIMP values are larger than those of NanoSIMS. In addition, the values of four elements are not expressed by a smooth curve, either Yb is depleted or Lu is enriched in SHRIMP data. Data are an average of eight measurements, so the shape is not derived from random effect. It is difficult to explain this irregularity by natural process and it may be due to experimental artifacts such as isobaric interference with a low mass-resolving power of ∼8,600 in Takehara et al. (2018), while NanoSIMS analyses were conducted under that of 9,400. This may be a reason for the discrepancy of Lu values between SHRIMP and NanoSIMS, but should be verified in a future work.

Figure 3B indicates the REE-pattern of QGNG zircon determined by a NanoSIMS (solid squares; our data) together with that measured by another SHRIMP at Hiroshima University (solid circles; Sano et al., 2002). These patterns are similar to those of AS3 zircons and consistent with the general signature of zircon stated above. When one takes into account of NanoSIMS light REE values such as La, Pr, Nd, Sm, and Eu except for Ce, they are systematically larger than those of SHRIMP, even though data are consistent with each other within 2σ experimental error. The isobaric interference is not the reason, because SHRIMP analysis was conducted under a resolving power of 9,300 in Hiroshima. These REE data were calculated by calibration against SL13 zircon standard, where REE abundance of SL13 was derived from Ireland and Wlotzka (1992). The original SL13 data were measured by a SHRIMP at the Australian National University with a low mass-resolving power and energy filtering method. They compared REE-patterns of SL13 and SL3, both derived from an alluvial deposit in Sri Lanka. Heavy REEs of SL13 were systematically one order of magnitude lower than those of SL3, while light REEs were somewhat irregular with a large error and much more depleted in SL13 (see Figure 4E of Ireland and Wlotzka, 1992). This underestimate of light REEs of SL13 may be the reason for the discrepancy between La, Pr, Nd, Sm, and Eu values of NanoSIMS and SHRIMP. Generally observed REE-patterns of AS3 and QGNG are very similar to those in the literature, suggesting a reasonable accuracy of NanoSIMS analyses within the experimental error margin of SIY.

### Partition Coefficients of REEs in Zircon/Melt

Because of their high charge and large ionic radii, REEs are considered to be incompatible elements during solidification of minerals such as quartz and plagioclase in felsic magma. REEs prefer to stay in silicate melt when the primary rock-forming minerals would crystallize. As a result, they are enriched in accessory minerals at the last stage of magma crystallization (Rollinson, 1993). Zircon is one of the accessory minerals and would concentrate selectively heavy REEs as shown in REE-pattern (see Figure 3). This preference of heavy elements is attributable to their smaller ionic radii, because light REE ions (REE^3+^) have relatively large ionic radius and cannot substitute for Zr^4+^ in the zircon lattice. This implication is further discussed by the Nernst partition coefficient, D_A_, defined as follows:

$${\text{D}}_{\text{A}}={\text{C}}_{\text{A}}^{\text{Zircon}}/{\text{C}}_{\text{A}}^{\text{Melt}}$$ (2)

where D_A_ is the partition coefficient for REE “A,” and C_A_^Zircon^ and C_A_^Melt^ are the concentrations of REE “A” in zircon and melt, respectively. It is possible to calculate D_A_, when REE data are available in host rock (groundmass as a hypothetical melt) and the mode of zircon is significantly small in the rock. For the case study, we measured REE abundance in zircon derived from Torihama dacite in Kyushu and listed in Table 4 together with those of whole rock from the literature. There is a data set of REE contents of Torihama zircon in Sano et al. (2002). To compare observed data with those by a SHRIMP, light REEs agree well with each other within 2σ error, while heavy REEs of NanoSIMS are systematically smaller than those of SHRIMP (see Supplementary Figure S4). In the case of QGNG comparison, light REEs of NanoSIMS are larger than those of SHRIMP, while heavy REES are consistent (Figure 3B). There is a possible grain-by-grain enrichment or depletion of overall REEs, that is, the shape of REE pattern is the same but moving vertically in the figure. If this is the case, the discrepancy of heavy REEs may be attributable to the underestimate of light REEs of SL13 by Ireland and Wlotzka (1992) as stated above.

**TABLE 4:** Rare earth elements concentrations in Torihama zircon and its host rock. Estimated partition coefficients together with ionic radius are also listed.

Element	Torihama zircon (ppm)	Whole rock^a^ (ppm)	Partition coefficient DA	Ionic radius^b^ (nm)
La	.019 ± .034	23.7	.0002 ± .0002	.1160
Ce	11.3 ± 3.0	42.3	.286 ± .085	.1143
Pr	.042 ± .012	4.18	.0104 ± .0045	.1126
Nd	.82 ± .14	15.0	.0570 ± .0137	.1109
Sm	3.29 ± .72	2.62	1.19 ± .355	.1079
Eu	.534 ± .096	.410	1.29 ± .264	.1066
Gd	14.6 ± 3.2	1.73	8.49 ± 1.91	.1053
Tb	6.28 ± 1.41	.335	19.5 ± 4.1	.1040
Dy	103.6 ± 24.0	2.43	44.2 ± 9.6	.1027
Ho	49.9 ± 12.2	0.572	87.9 ± 22.5	.1015
Er	260.7 ± 62.6	1.77	148 ± 37.6	.1004
Tm	77.2 ± 18.6	.289	261 ± 77	.0994
Yb	792.9 ± 171.5	2.00	385 ± 106	.0985
Lu	160.3 ± 28.0	.390	403 ± 86	.0977

a Ce, Nd, Sm, Eu, Gd, Er, Yb and Lu were from Nagasawa (1970) and other elements were estimated by Sano et al. (2002).

b From Shannon (1976).

**FIGURE 4:**
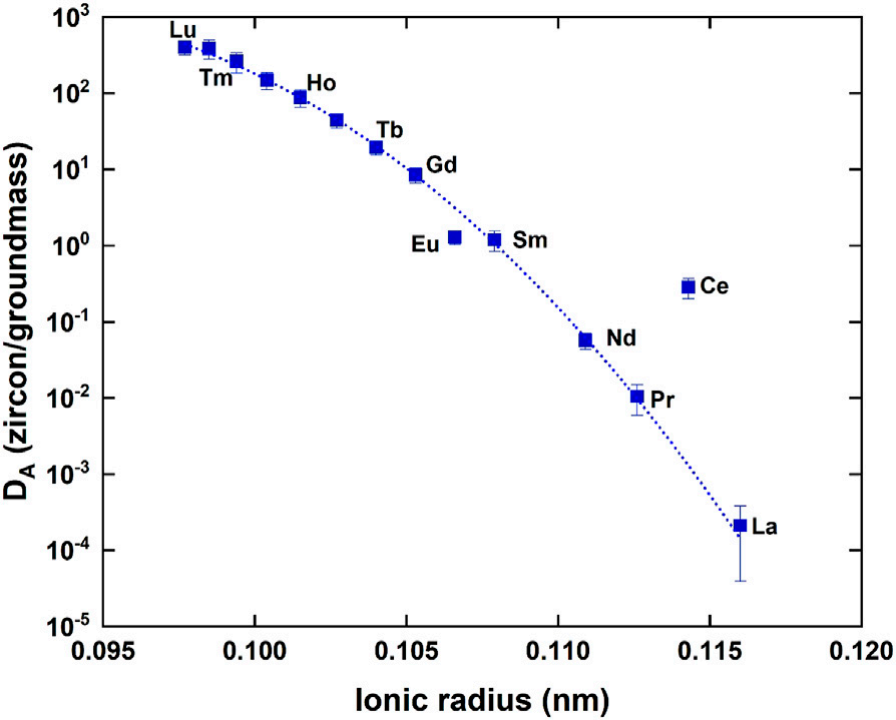
A correlation diagram between the ionic radius of rare earth element and the partition coefficient (DA) between zircon and melt. Error assigned to the symbol is 2σ. A dotted curve shows a best fit of Eq. 3 by a least-squares method where Ce and Eu data are masked.

Based on Eq. 2, we calculate partition coefficients (D_A_) of zircon-melt for all REEs and listed in Table 4. Errors assigned to D_A_ are 2σ derived from those of REE contents in Torihama zircon. Estimated D_A_ values vary significantly from .0012 of La to 236 of Lu, covering almost five orders of magnitude with an atomic number. This great variation is well explained by a compatibility of REE ionic radius with Zr ion as stated above. A simple parabolic shape was found between ionic radius and logarithm of partition coefficient in a crystal-melt system (Onuma et al., 1968). This is due to the optimal site size for the element that is substituted in crystal lattice and applicable to the zircon-melt partitioning (Hanchar and Van Westrenen, 2007). Figure 4 shows a relationship between ionic radius and D_A_ value of all REEs obtained in this work. D_A_ value is decreasing smoothly and monotonically with its ionic radius except for apparent outliers of Eu and Ce. This is possibly showing a right side half of the parabola. It is necessary to explain the relation more theoretically.

Blundy and Wood (1994) reported a rational elastic moduli model to explain the portioning behavior of cations between crystal and melt and presented the following formula:

$${\text{D}}_{\text{A}}{=\text{D}}_{\text{0}}\text{×exp}\left[-\alpha \text{×}\left\{{\text{r}}_{\text{0}}/2\text{×}{\left({\text{r}}_{\text{A}}{-\text{r}}_{\text{0}}\right)}^{2}+1/3\text{×}{\left({\text{r}}_{\text{A}}{-\text{r}}_{\text{0}}\right)}^{3}\right\}\right]$$ (3)

where D_0_ and r_0_ denote the optimal partition coefficient (maximum of the parabola) and optimal ionic radius, respectively. α is a constant under the given pressure, temperature, and chemical compositions. r_A_ is the ionic radius of interest REE “A”. Curve fitting of D_A_ value is made by a least-squares method under masking of Eu and Ce, because they may have different valences such as Ce^4+^ and Eu^2+^. A best fit gives D_0_ = 1,499 ± 377, α = (4.49 ± 0.39) ×10^5^, and r_0_ = .0901 ± .0009 where errors are 2σ, R^2^ = .992 and MSWD = .12. It is noted that the estimated r_0_ value agrees well with the radius of Zr^4+^, .084 nm in zircon crystal. There may be Ce^4+^ ions in the zircon lattice, whose ionic radius is .097 nm, much smaller than 0.1143 nm of Ce^3+^ and close to the optimal value. This possibly makes the D_A_ value larger than that expected by Eq. 3. Similar explanation is applicable to D_A_ value of Eu, because Eu^2+^ has a larger ionic radius of .125 nm than .1066 nm of Eu^3+^. These lines of evidences support the robustness of the elastic moduli model by Blundy and Wood (1994).

### Equilibrium Melt Estimation of REEs

It is possible to reproduce bulk rock REEs contents by using D_A_ values and detrital zircon REEs, where there is no information available on the parent rock composition. This is called “back-calculation” (Hoskin and Black, 2000; Hanchar and Van Westrenen, 2007), which is also useful to evaluate D_A_ values in the literature by comparing the reconstructed REE pattern with that of original whole rock. A new method of such reconstruction (Chapman et al., 2016) showed a negative correlation between D_A_ values and “A” content, especially in the case of light REEs such as La. Anyhow, we compare the present D_A_ values with those of Hinton and Upton (1991), Sano et al. (2002), and Chapman et al. (2016). The data set of Hinton and Upton (1991) is one of the most cited references in the geochemistry field. That of Sano et al. (2002) was recommended by Hanchar and Van Westrenen (2007) where their data set conformed best to the elastic moduli model of Blundy and Wood (1994). The new method of Chapman et al. (2016) may be a more reliable estimate of D_A_ among recent studies because they treated carefully experimental data together with those in the literature. Even though D_A_ values of light REEs in Hinton and Upton (1991) are apparent outliers, a wide range of variations up to six orders of magnitude exists in the diagram (Supplementary Figure S5). Thus, it is difficult to make a direct comparison of our data set with others. In addition, the Sm value of Chapman et al. (2016) is somewhat larger than others.

**FIGURE 5:**
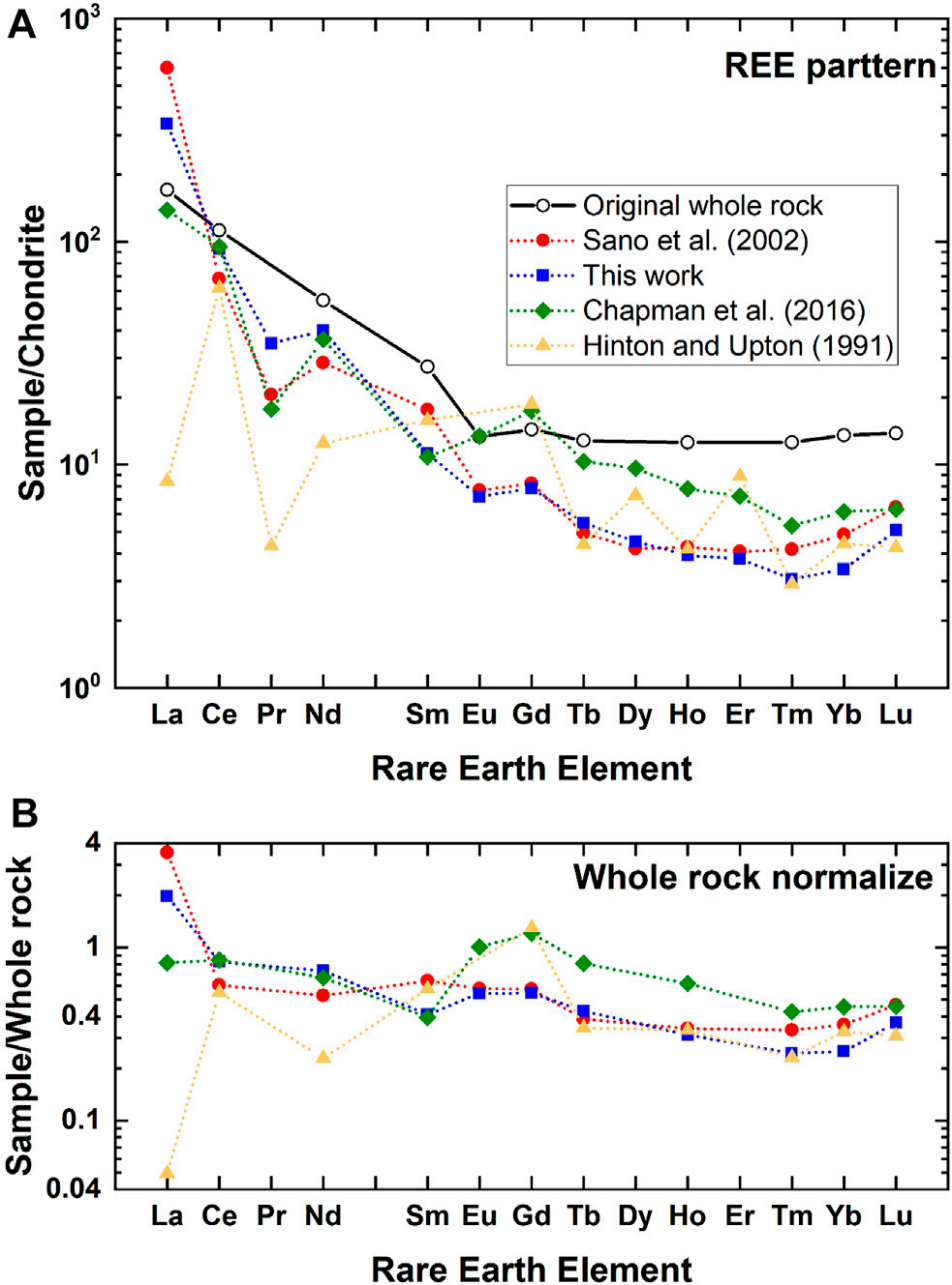
**(A)** Chondrite normalized rare earth elemental abundances of original whole rock (BP11) and those of estimated melt by a back-calculation using partition coefficients of this work and those from references. **(B)** Whole rock normalized rare earth elemental abundances of estimated melt by a back-calculation.

In order to make a back-calculation, we have prepared a data set of REEs in zircon and its host rock (BP11), adamellite, and granodiorite in the Boggy Plain zoned pluton located in eastern Australia (Hoskin and Black, 2000) because they were already adopted in Sano et al. (2002) and Hanchar and Van Westrenen (2007). Figure 5A shows REE-patterns of reconstructed melt for BP11 by D_A_ estimated in this work together with those of referenced data (Hinton and Upton, 1991; Sano et al., 2002; Chapman et al., 2016). Original whole rock data are also described in the diagram. To compare these values more visually, REE data were further normalized against those of whole rock and indicated in Figure 5B. Light REEs such as La and Nd are variable, possibly due to their low abundances in zircon and the negative correlation between D_A_ and REE contents (Chapman et al., 2016). Taking a series of REEs from Nd to Lu with an atomic number, all reconstructed REE patterns resemble those of original whole rock. To watch the shape of pattern more precisely, data of this work and Sano et al. (2002) are flat in a horizontal manner, while the Chapman et al. (2016) and Hinton and Upton (1991) data show significant variations. The averages and its standard deviations of normalized values for this work and that of Sano et al. (2002) are .62 ± .11 and .47 ± .23 (both 2σ errors), respectively. These variations are smaller than those from Chapman et al. (2016) and Hinton and Upton (1991) (.67 ± .57 and .46 ± .71, respectively). These analyses suggest that the D_A_ values in the present work are the best estimate of partition coefficients of REE in the zircon/melt system except for those of La and Ce.

## Conclusion

Rare earth elements (REEs) are essential tracers in high temperature geochemistry such as fractional crystallization of magma. An analytical procedure for all REE contents in silicate glass and zircon has been developed by a NanoSIMS with a 7–8-μm diameter probe and high mass-resolving power of 9,400. The overall sensitivity and detection limit are approximately 8 cps/ppm/nA and 1 ppb, respectively, which indicate reasonable performance by the SIMS instrument. Secondary ion yields of REEs in the SRM610 standard glass are identical to those in 91500 zircon standard, suggesting that it is possible to use a glass standard to calibrate against during the measurements of REEs in zircon samples. Best partition coefficients of REEs in the zircon/melt system are estimated by analyses of zircon in a Quaternary magmatic system. Their relationship with the ionic radius of REEs is well explained by an elastic moduli model. Their significance is verified by a back-calculation, reproducing the original melt REEs pattern. Considering the achieved spatial resolution, the method may be applicable to evaluate elemental gradients in some samples by a fundamental process.

## Data Availability

The original contributions presented in the study are included in the article/Supplementary Material, further inquiries can be directed to the corresponding author.
