# Supplementary material for: NanoSIMS Analysis of Rare Earth Elements in Silicate Glass and Zircon: Implications for Partition Coefficients
Source: Front Chem. 2022 Mar 14;10:844953. doi: 10.3389/fchem.2022.844953 (PMC8963819; doi:10.3389/fchem.2022.844953)
Supplement: Supplementary file 1 [file DataSheet1.PDF]

**Supplementary Materials for**

# NanoSIMS analysis of rare earth elements in silicate glass and zircon: Implications for partition coefficients

**By Shi et al.**

**Supplementary Figures**

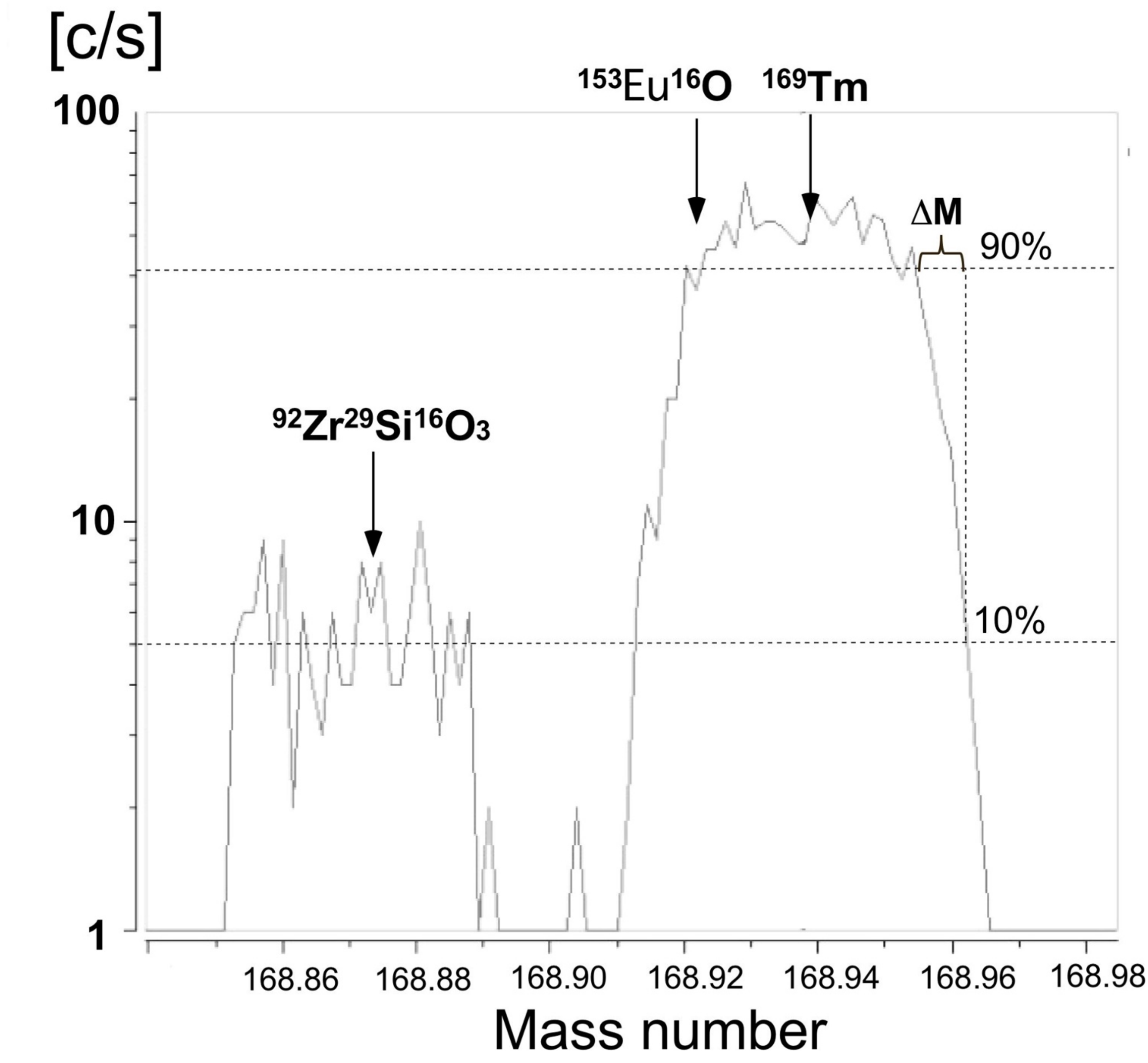


**Supplementary Fig. 1**

Mass spectrum of $^{169}Tm$, $^{153}Eu^{16}O$ and $^{92}Zr^{29}Si^{16}O_3$ of SRM610 glass standard in a vicinity of mass number 169 measured by a NanoSIMS. ΔM shows a shoulder width of 10%-90% peak height.

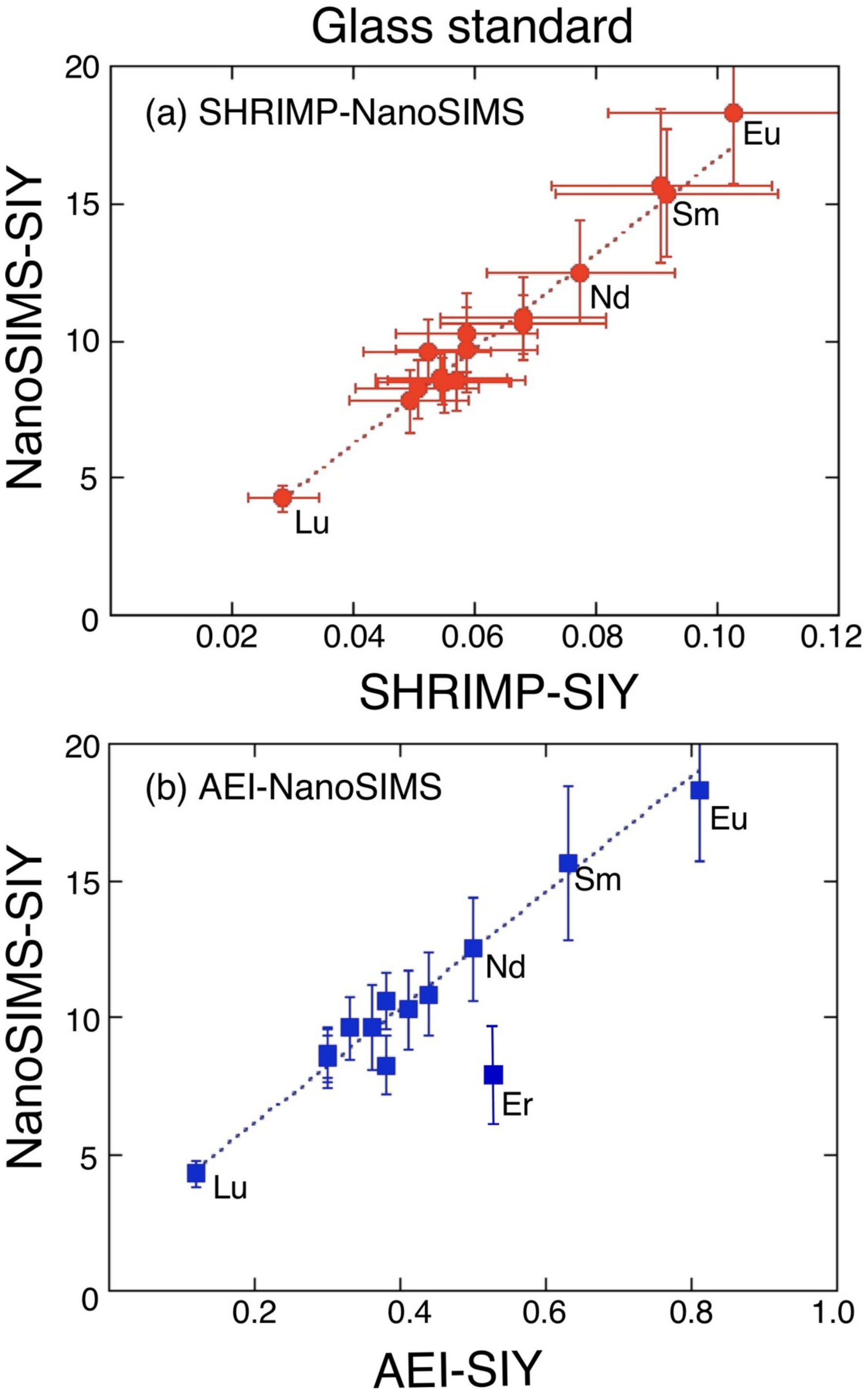


**Supplementary Fig. 2**

A correlation diagram between secondary ion yields (SIYs) of rare earth elements in glass standard measured by SHRIMP (Sano et al., 2002) and NanoSIMS (This work). Error assigned to the symbol is two sigma. A dotted line shows a best fit. (b) A diagram between SIYs of AEI instrument (Reed, 1983) and NanoSIMS (This work).

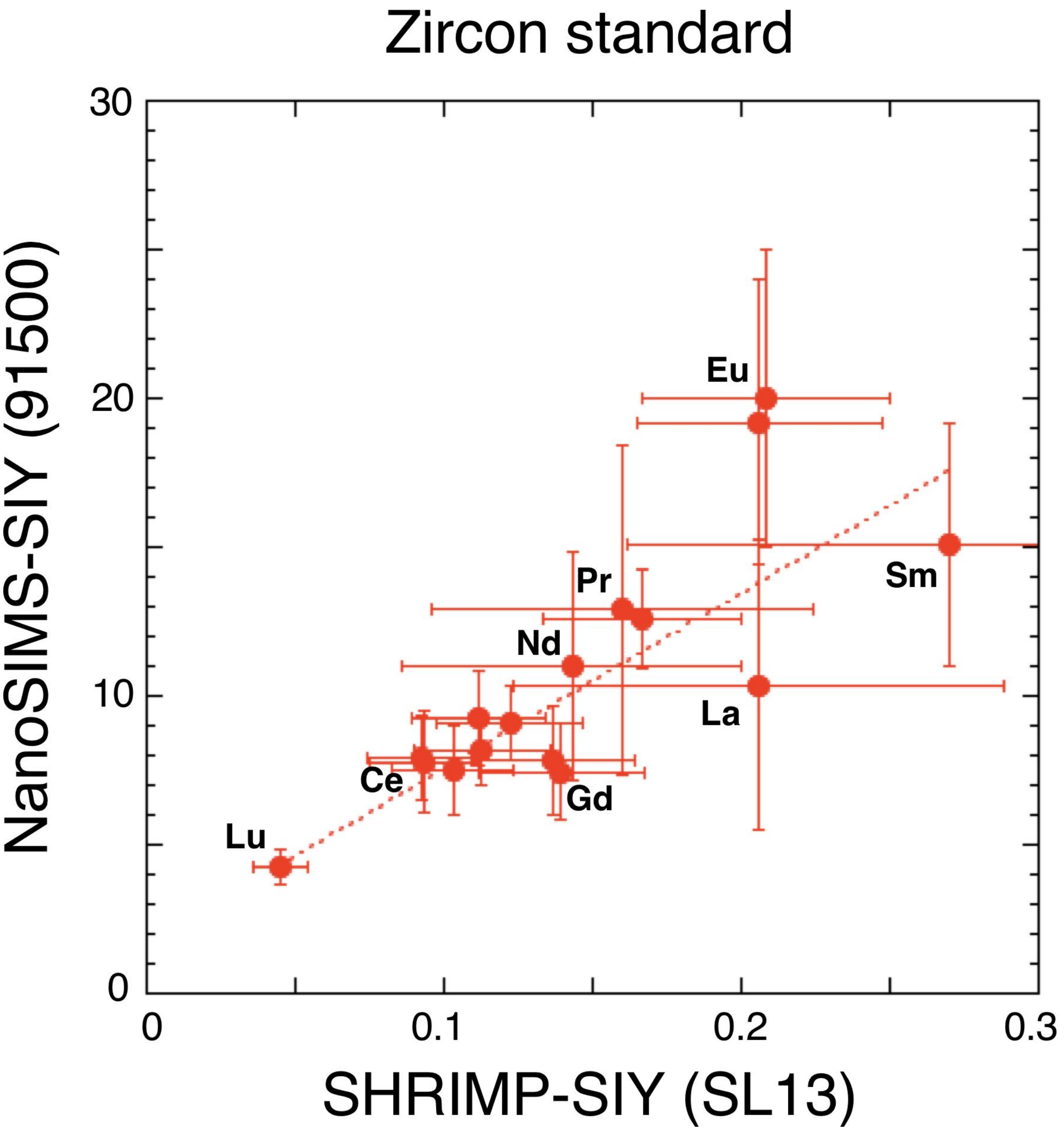


**Supplementary Fig. 3**
A correlation diagram between secondary ion yields (SIYs) of rare earth elements in zircon standard measured by SHRIMP (Sano et al., 2002) and NanoSIMS (This work). Error assigned to the symbol is two sigma. A dotted line shows a best fit.

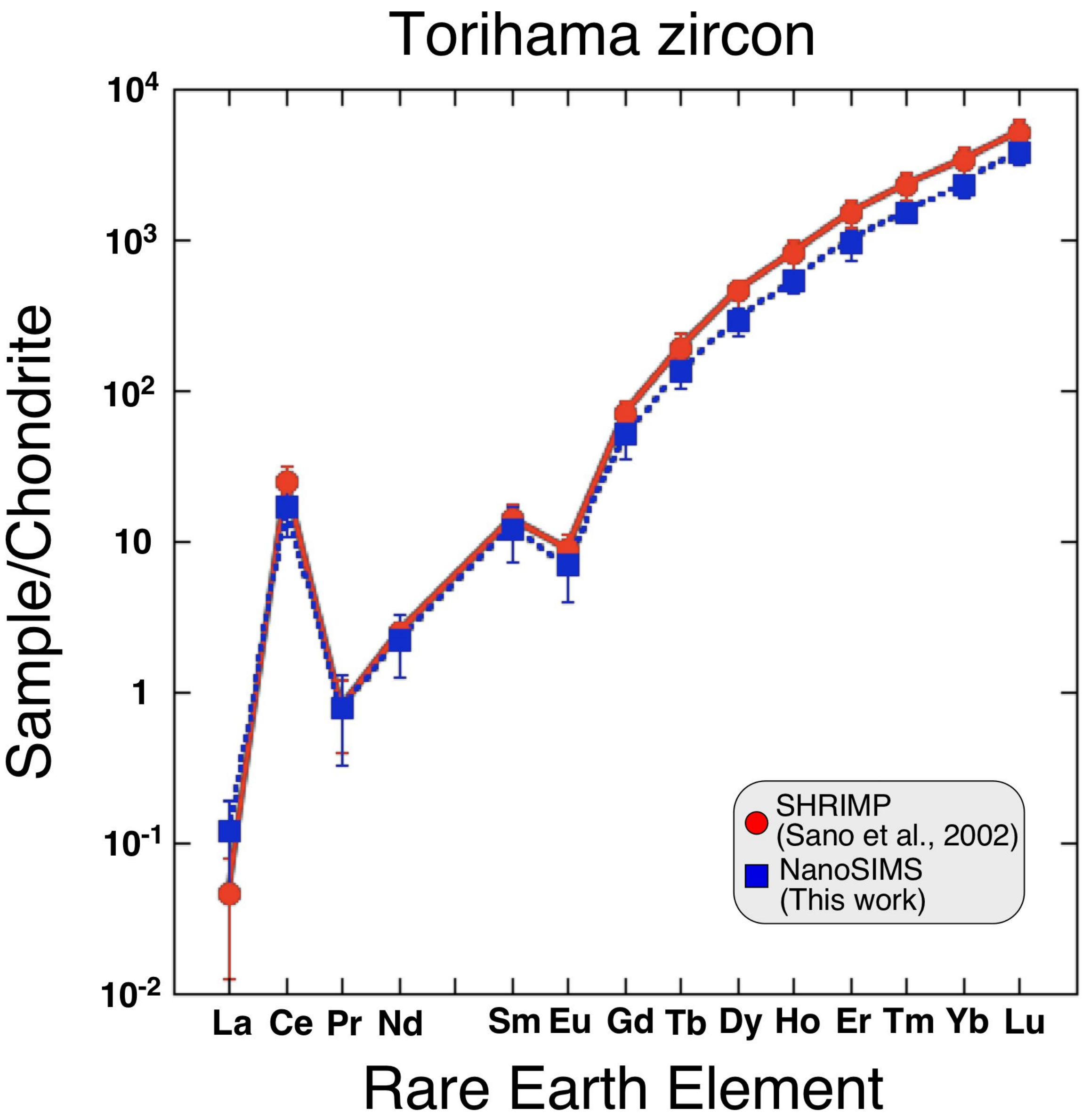


**Supplementary Fig. 4**

Chondrite normalized rare earth elemental abundances of the Torihama zircon. Solid circle and square show data of SHRIMP (Sano et al., 2002) and those of NanoSIMS (This work). Error assigned to the symbol is two sigma.

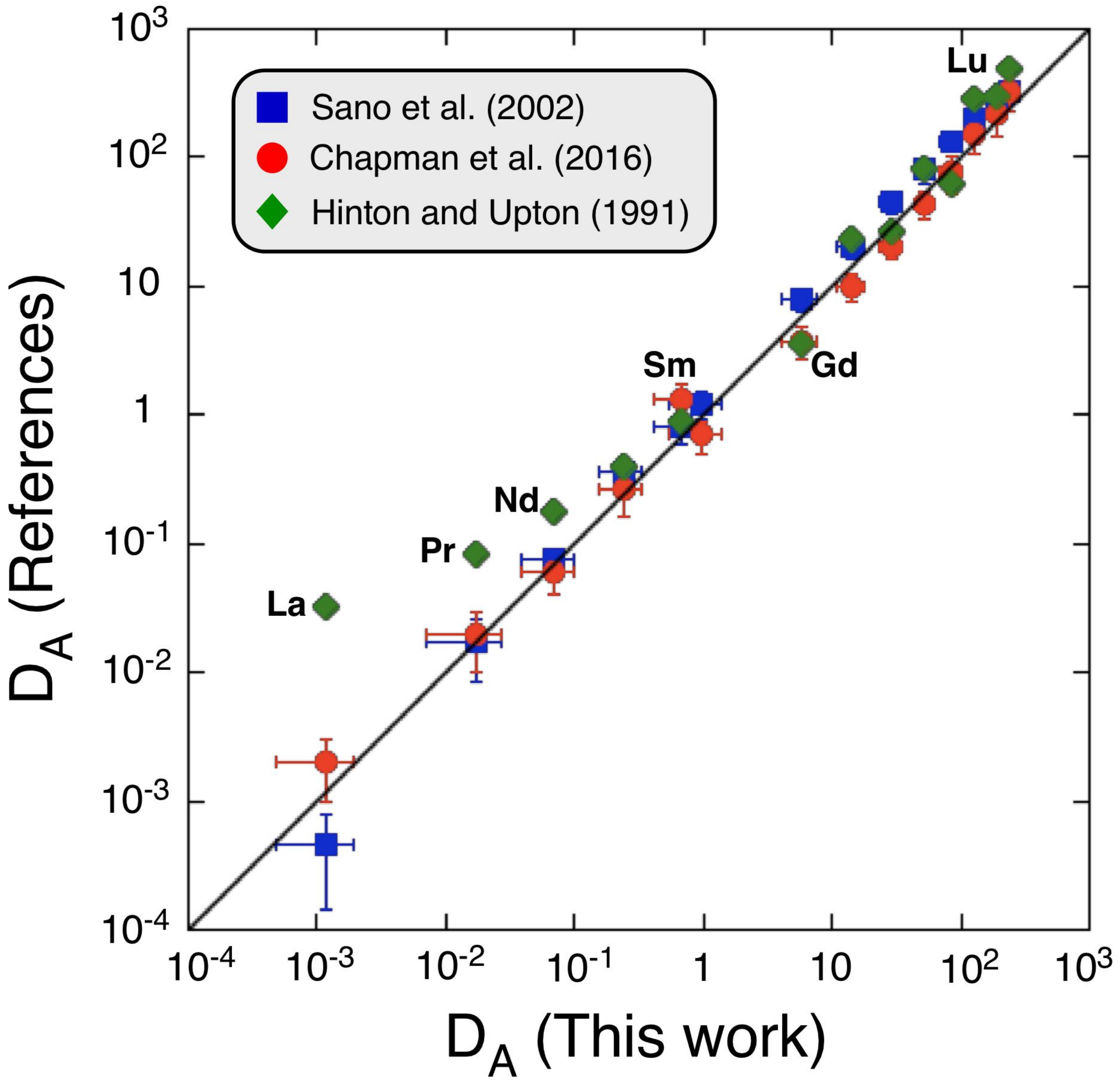


**Supplementary Fig. 5**
A correlation diagram between partition coefficients of rare earth elements between those in this work and those in references (Sano et al., 2002; Chapman et al., 2016; Hinton and Upton, 1991). Error assigned to the symbol is two sigma. There is no error available in Hinton and Upton (1991).
